# The Additional Accuracy Gained by Cone Beam CT in Shape-Sensing Robotic Bronchoscopy

**DOI:** 10.1016/j.chpulm.2025.100203

**Published:** 2025-08-07

**Authors:** Alberto E. Revelo, Jing Peng, Jianing Ma, Michael Woods, Christian Ghattas, Jasleen Pannu, Jeffrey C. Horowitz, Nicholas Pastis

**Affiliations:** a Department of Pulmonary, Critical Care and Sleep Medicine, The Ohio State University Wexner Medical Center and The James Comprehensive Cancer Center, Columbus, OH; b Center for Biostatistics, The Ohio State University Wexner Medical Center, Columbus, OH

**Keywords:** cone beam CT scan, guided bronchoscopy, lung cancer, lung biopsy, pulmonary nodules, radial endobronchial ultrasound, robotic-assisted bronchoscopy, tool-in-lesion

## Abstract

**Background:**

Cone beam CT (CBCT) scan provides an intraprocedural 3-dimensional image of the biopsy tool in relation to the lesion, which may enhance the accuracy of robotic-assisted bronchoscopy (RAB) through real-time bronchoscopy adjustments. Our goal is to evaluate the ability to accurately position, in real time, a biopsy needle in the center of a pulmonary nodule using shape-sensing RAB alone vs shape-sensing RAB + CBCT guidance.

**Research Question:**

Does the addition of CBCT improve the accuracy of robotic bronchoscopy?

**Study Design and Methods:**

A total of 102 nodules were biopsied using shape-sensing RAB and the position of the needle in relation to the center of the nodule identified using a ceiling-mounted CBCT scanner. Repositioning of the RAB after 1 or 2 CBCT adjustments was accomplished using information gathered from the 3-dimensional images and using an updated augmented fluoroscopy target. The primary end point was needle location and distance change of the needle tip in reference to the lesion center using RAB alone vs RAB + CBCT scan. Secondary end points were improvement in radial endobronchial ultrasound image and average number of CBCT spins required to land at the center of the target.

**Results:**

Using RAB alone, the needle was placed in the center in 27 nodules (26.5%). The addition of CBCT scan to RAB greatly improved the distance of the needle toward the center of the lesion (mean ± SD, −4.08 ± 4.63 mm) in 46 nodules (61.3%) after 1 CBCT adjustment and (mean ± SD, −4.02 ± 4.21 mm) in an additional 17 nodules (58.6%) after a second CBCT adjustment (*P* < .001). Radial endobronchial ultrasound image was also improved from eccentric to concentric.

**Interpretation:**

The addition of CBCT scan to RAB was shown to improve the position of a biopsy tool in relation to the center of a pulmonary nodule. To our knowledge, this study is the first to quantify the accuracy achieved using both technologies, which may translate into reliability to perform diagnostic and potentially future therapeutic interventions in guided bronchoscopy.

**Take-Home Points**

**Study Question:** Our study questions are as follows: (1) does adding cone beam CT (CBCT) scan to robotic-assisted bronchoscopy (RAB) improve accuracy of tool placement in the center?; (2) what are the factors that influence center strike?; and (3) what is the average number of spins required to hit the center of a nodule?

**Results:** Adding CBCT scan to RAB was shown to improve accuracy of tool placement in the center. Radial endobronchial ultrasound view was strongly associated with the ability to hit the center of a nodule. An average of 2 CBCT spins were required to place a tool in the center.

**Interpretation:** The accuracy of placing a tool in the center using RAB + CBCT scan may be key to therapeutics. Radial endobronchial ultrasound images were improved with CBCT adjustments. Cumulative dose area product and cumulative air kerma were similar when CBCT scan was used to adjust a tool in the center of a nodule.

With increased utilization of lung cancer screening and an increase in incidental nodule detection, pulmonary nodules are discovered in approximately 1.6 million people per year in the United States.^1^ Lung cancer is the leading cause of cancer mortality in the United States and worldwide, and although > 95% of pulmonary nodules are benign,^2^ the discovery of a nodule leads to concern among clinicians, patients, and families.^3^ Diagnostic sampling is typically performed for intermediate- and select high-risk nodules via transthoracic needle aspiration (TTNA), surgery, or navigational bronchoscopy. Guided bronchoscopy is safer than TTNA and surgery^4^ and provides an opportunity for mediastinal staging with endobronchial ultrasound transbronchial needle aspiration in the same procedural setting.

Published studies show that the diagnostic yields of guided bronchoscopy have vastly surpassed those of conventional bronchoscopy,^5^ but they have not consistently reached the published yields of TTNA.^6^ To improve accuracy, robotic-assisted bronchoscopy (RAB) has been added to navigational bronchoscopy platforms; however, despite the incorporation of RAB, published diagnostic yields have not significantly changed.^7^ Potential explanations for plateauing yields include inadequate sampling tools and the lack of an accurate real-time confirmatory technique of biopsy tool in lesion. The most commonly used confirmatory technique, radial endobronchial ultrasound (rEBUS), may provide false-positive images because of atelectasis or intraprocedural hemorrhage.^8^^,^^9^ An additional barrier to guided bronchoscopy lies in the time between the preprocedural mapping CT scan and the procedure. Moreover, although the planning CT scan is acquired during full inspiration in an awake patient, the procedure is done in patients on mechanical ventilation with volumes that do not match full inspiration, leading to changes in lung anatomy and nodule positioning. This results in CT-to-body divergence, which can lead to a divergence between the expected and actual location of the target lesion.^10^

Cone beam CT (CBCT) imaging has emerged as a potential solution to improve diagnostic accuracy of guided bronchoscopy by overcoming CT-to-body divergence.^8^^,^^11^^,^^12^ CBCT scan provides an intraprocedural 3-dimensional (3D) image of the biopsy tool in relation to the lesion, which may enhance the accuracy of RAB through real-time bronchoscopy adjustments.^13^ Furthermore, bronchoscopic ablation is considered a potential therapeutic intervention, and CBCT scan has been proposed as a critical tool to verify that a tool is safely and accurately positioned.^14^ While a diagnosis can be made without a tool reaching the center of the lesion, centering a tool may be used as a marker of accuracy for a technology. In addition, if the future of navigational bronchoscopy includes ablation, then accurate placement of a tool within a lesion has increased importance when planning ablation zones and accounting for important neighboring structures.^15^^,^^16^ We undertook this trial to evaluate the ability to accurately position, in real time, a biopsy needle in the center of a pulmonary nodule using RAB alone vs RAB + CBCT guidance. In our study, we define the primary end point, accuracy, as the ability to truly place the needle in the center of a nodule, which is different from diagnostic accuracy, the ability to rule-in or rule-out a specific disease state. When it comes to peripheral therapeutics, being accurate within a target will be a crucial step to successful treatment because it confirms the position with CBCT scan of an instrument in relationship to the lesion. On the other hand, it is not necessary to be centered within a lesion to improve diagnostic accuracy because being off-center or in the peripheral portion of a nodule is often sufficient to obtain a diagnosis.

## Study Design and Methods

### Participants

This is a prospective, single-center, observational study of 110 consecutive patients meeting inclusion criteria who were scheduled for RAB biopsy. Institutional review board approval was granted under The Ohio State University (No. 2022C0129). Patients ≥ 18 years of age with detected pulmonary nodules ≤ 30 mm in diameter were eligible for study enrollment. Patients who met the aforementioned definition were screened using the inclusion and exclusion criteria. The CT images were reviewed by the investigators to determine the size, location, and radiographic characteristics of the nodules. Patients were excluded from participation if they were pregnant, could not undergo bronchoscopy, declined to participate, or were unable to consent for the study.

### Bronchoscopic Procedure

After informed consent was obtained, patients were placed on the procedure table in a supine position. An anesthesiologist induced sedation and neuromuscular blockade in all cases to perform endotracheal intubation with a ≥ 8.5-mm inner diameter endotracheal tube. The following mechanical ventilation parameters were immediately applied as clinically tolerated: positive end-expiratory pressure ≥ 10 cm H_2_O, tidal volume between 8 and 10 mL/kg ideal body weight, and Fio_2_ ≤ 0.4. A flexible bronchoscopy with suctioning was initially performed on all patients. A ceiling-mounted C-arm system with CBCT capabilities (Philips Allura FD20; Philips) was used with all patients. The robotic catheter was advanced into the trachea, and the navigation start time was recorded. The navigation time was complete when the scope was as close as possible to the target within reach of a 3-cm needle while the target was centered on the shape-sensing robotic-assisted bronchoscopy (ssRAB) screen. rEBUS was used for confirmation of nodule position when possible and included in navigation time.

**Figure 1:**
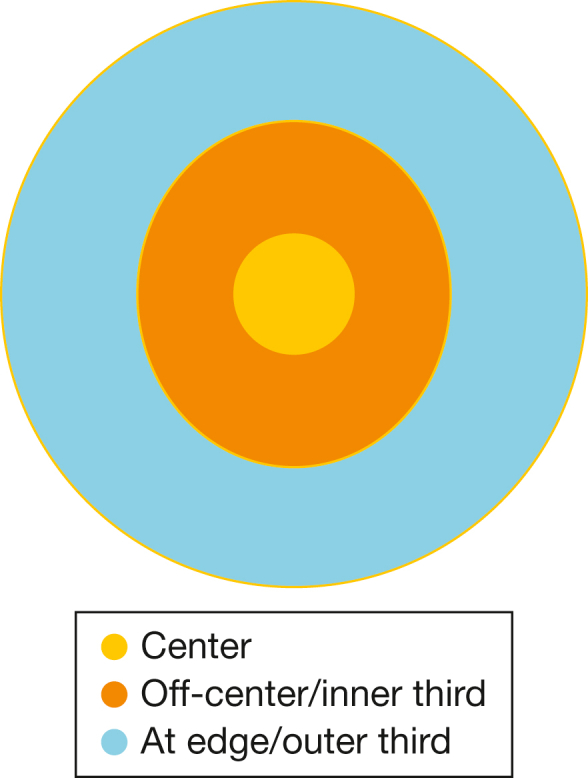
Schematic visualization of a target showing center, off-center/inner third, and at edge/outer third.

Before needle deployment, the target was aligned so the cross-mark was at the center of the preplanned target. An rEBUS probe was used to interrogate all lesions before biopsy and as needed. rEBUS signals were recorded as concentric, eccentric, or no image. A biopsy needle (Flexision, Intuitive Surgical Inc) was used for initial sampling of the target and for all measurements recorded from CBCT images. Other biopsy tools were used per physician discretion after completion of needle sampling and measurements. After the first needle stick, an inspiratory breath hold with an adjustable pressure-limited valve at 25 cm H_2_O was performed for initial and subsequent CBCT spins. 3D images were reviewed by the clinician, the lesion was segmented, and the position of the needle was recorded. Both qualitative and quantitative variables were used to describe our primary end point, wherein recording distance from center in millimeters and visual description of the needle in relationship to the lesion was used (eg, off-center, at edge, and outside of the lesion) (Fig 1). If the needle was not in the center of the target, the robot was repositioned according to information provided by the initial CBCT scan obtained. This was followed by a second needle pass and CBCT spin to reassess needle position after an adjustment. Up to 2 CBCT adjustments were performed per lesion with a maximum of 3 images obtained (initial CT scan, followed by first and second CBCT adjustments as applicable). Not all patients needed > 1 CBCT adjustment.

**Figure 2:**
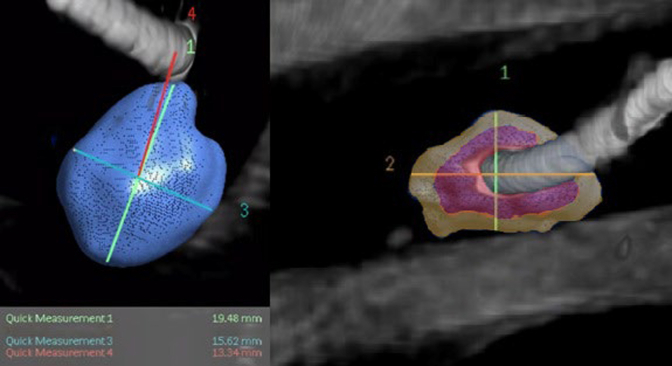
A, Quantitative assessment of tool distance from center of the target. Using this image obtained from cone beam CT scan, the center of the nodule is found (intersection between green and teal lines). A measurement is then obtained from the center of the target to the center of the catheter (red line) to determine the distance from the center; in this case, it is 13.34 mm (or 4.45× catheter diameter). The needle is not seen here because it has been deployed away from the viewer. All nodule measurements were obtained with the same method. B, Qualitative assessment of a tool in relationship to the center of a nodule. The catheter is in the center of the target. The image is divided in thirds with the pink zone representing the center, the purple zone representing the off-center or inner third, and the outer brown zone representing the at edge or outer third.

Repositioning of the RAB after 1 or 2 CBCT adjustments was accomplished using information gathered from the CBCT 3D images and using an updated augmented fluoroscopy target. The amount of movement required to reposition was estimated by knowing the outer diameter of the RAB catheter (3.5 mm) along with distance from center measurements. For instance, if distance from the center was about 9 mm, we would move the equivalent of 3 catheter widths under live fluoroscopy. An augmented fluoroscopy image allows for this type of real-time readjustment (Fig 2).

### Data Analysis

The sample size estimation was based on previously published data on accuracy wherein RAB can reach a lesion in 80% of the cases.^17^ We expected that the ability to localize a lesion with RAB would be increased by 10% with CBCT scan. Based on the power calculation using a paired *t* test assuming the same SDs with 80% power and a significance level of 0.05, we estimated that 85 participants would be needed to detect a 10% difference with effect size as little as 0.3. The power calculation was performed using PASS 2020 (version 20.0.6; NCCS, LLC).

The primary end point was needle location and distance change of the instrument tip in reference to the lesion center using RAB alone vs RAB + CBCT scan. Secondary end points included rEBUS image improvement (ie, from no image to image, from eccentric to concentric) and average number of CBCT spins required to land at the center of the target. To make a comparison with respect to the clinically meaningful distance to the lesion center, we categorized the needle location in 4 ordinal groups: center, off-center, at edge, and outside of the target. The needle distance change was defined as the difference between the needle distance with RAB + CBCT scan and the needle distance with RAB alone ([RAB + CBCT scan] – RAB). Because the difference represents movement toward the center (0 mm) and because it is desirable for RAB + CBCT scan to be the smaller number, a negative number means a favorable change.

Patient characteristics were summarized using descriptive statistics. Categorical variables were reported as frequencies (%). Continuous variables were reported as mean ± SD if they were approximately normally distributed; otherwise, median with interquartile range (25th-75th percentile) were reported. To analyze the needle location change and rEBUS image view, Wilcoxon signed rank test was used to compare the needle location between RAB alone vs RAB + CBCT scan. To analyze the needle distance difference between RAB alone vs RAB + CBCT scan, a paired *t* test was used. Additionally, we performed univariate logistic regression analysis of factors that could affect associations between center strike detection and risk factors. The statistical analysis was performed using R software (version 4.3.0; R Core Team, R Foundation for Statistical Computing).

## Results

Of 114 patients consecutively enrolled, 98 patients met criteria for the study and a total of 102 nodules were included in the analysis. The mean age was 66 years; patients included 54 female patients (53.5%), and most were White (90.1%) (Table 1).

**Table 1:** Patient Demographics (N = 98)

Variable	Value
Age, y	66.5 [9.30]
Sex	
Female	52 (53.1)
Male	46 (46.9)
Race	
American Indian or Alaska Native	0 (0)
Asian	3 (3.1)
Black or African American	6 (6.1)
Native Hawaiian or Other Pacific Islander	0 (0)
White	89 (90.8)
BMI, kg/m^2^	28.63 [6.94]
Smoking status	
Active	25 (25.5)
Prior	50 (51.0)
Never	23 (23.5)
Comorbidities	
History of lung cancer	17 (17.3)
Asthma	19 (19.4)
COPD	40 (40.8)
CAD	22 (22.4)
Heart failure	11 (11.2)
Hypertension	61 (62.2)
Diabetes mellitus	29 (29.6)

Data are presented as No. (%) or mean [SD]. CAD = coronary artery disease.

The majority of the lung nodules were in the upper lobes on CT scan; 49.0% of them had a bronchus sign and 71% had a vessel sign (ie, artery leading directly to nodule on CT scan) (Table 2). The median time from navigation start to first CBCT spin was 4 minutes.

**Table 2:** Nodule Characteristics (n = 102)

Characteristic	Value
Size, mm	15.5 (12.0-18.0)
Location	
Right upper lobe	28 (27.5)
Right middle lobe	9 (8.8)
Right lower lobe	23 (22.5)
Left upper lobe	28 (27.5)
Left lower lobe	14 (13.7)
Composition	
Solid	96 (94.1)
Part-solid	6 (5.9)
Ground glass	0 (0)
Shape	
Round/smooth	40 (38.1)
Lobulated	30 (28.6)
Spiculated	32 (30.5)
Other	3 (2.9)
Presence of calcification	6 (5.9)
Distance from pleura on axial view, mm	15 (0-24)
Bronchus sign	50 (49.0)
Airway < 1 cm from nodule (on axial view)	74 (72.5)
Vessel sign	73 (71.6)

Data are presented as No. (%) or median (interquartile range).

**Figure 3:**
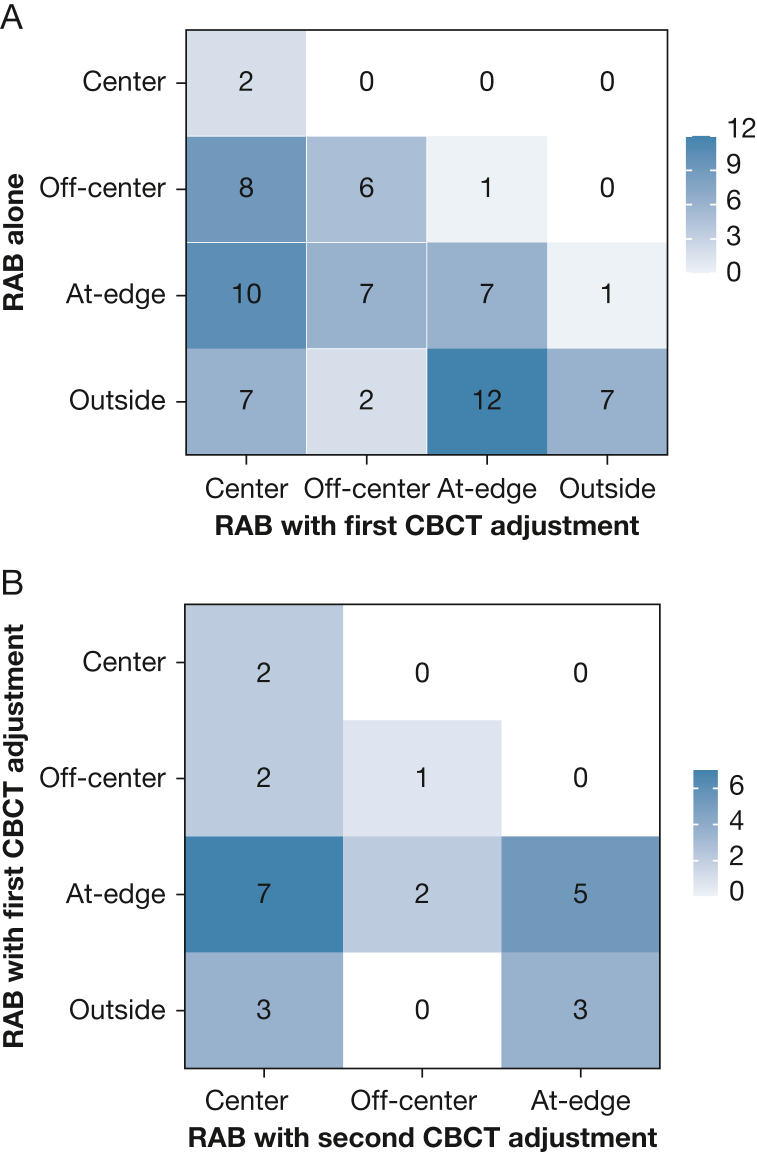
A, Needle location heat map: RAB alone vs RAB with first CBCT adjustment. Position closer to the center improved in 46 nodules after the first CBCT adjustment. No adjustment was made when at-center with RAB alone. B, Needle location heat map: RAB with first CBCT adjustment vs RAB with second CBCT adjustment. Position closer to the center improved for an additional 17 nodules after a second CBCT adjustment. CBCT = cone beam CT; RAB = robotic-assisted bronchoscopy.

When comparing robotic bronchoscopy alone vs robotic bronchoscopy with first CBTC adjustment, the addition of CBCT scan improved the needle position toward the center of the lesion for a total of 46 nodules, as shown in Figure 3A, and this was statistically significant (*P* < .001). With the addition of a second CBCT adjustment, we noticed improvement in position closer to the center for an additional 17 nodules, and this was also statistically significant (Fig 3B).

**Figure 4:**
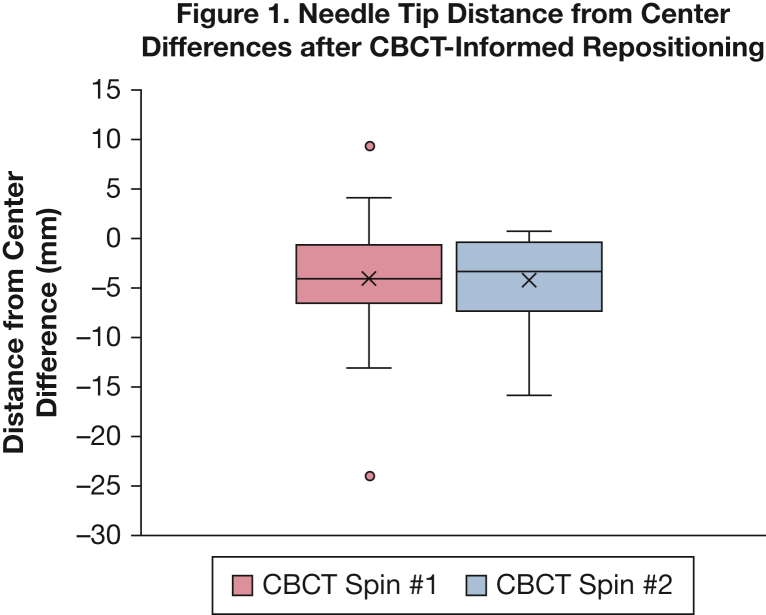
Distribution of these measures in a boxplot. Note that negative values of change in distance (Δd) are favorable because they indicate the needle being closer to the center of the lesion after repositioning. Using paired *t* test, the distance from the center was found to be improved by 4.08 ± 4.63 mm after CBCT spin 1-informed adjustment and improved for most nodules after CBCT spin 2-informed adjustment (*P* < .001). CBCT = cone beam CT.

To identify improvements in the positioning of the needle tip after CBCT-informed adjustments, the difference in distance from center (mm) was calculated as follows:

$$\Delta d_{CBCT\#1}=d_{\left(RAB+CBCT\#1\right)}-d_{\left(RAB\;Alone\right)},\Delta d_{CBCT\#2}=d_{\left(RAB+CBCT\#1+CBCT\#2\right)}-d_{\left(RAB+CBCT\#1\right)}$$

and results were shown in the boxplot distribution of difference in the needle distance both for RAB with first CBCT adjustment – RAB alone and RAB with second CBCT adjustment – RAB with first CBCT adjustment (Fig 4). A negative number means a favorable change with the tool moving toward the center, indicating the needle distance is improved for most of the nodules after these adjustments.

Thirty nodules were determined to have a needle location of outside with RAB alone. Twenty-eight of these nodules underwent first CBCT adjustment. For these nodules, the median difference in distance from center was −4.20 mm (−7.47, −4.20 mm).

We had 102 nodules with a diagnostic outcome. The strict diagnostic yield was 70%, and the intermediate diagnostic yield was 73%. The diagnostic yield for malignancy was 91%. The intermediate diagnostic yield calculation incorporated longitudinal follow-up imaging data at 12 months. Two patients were lost to follow-up, and another patient has not yet completed the 12-month follow-up period. All procedures were performed at a medical center located in an endemic histoplasmosis region. Additional information regarding diagnostic yield in RAB alone vs RAB + CBCT can be found in Supplemental material e-Figure)

## Discussion

Although novel robotic bronchoscopy platforms combined with 3D imaging systems are becoming mainstream diagnostic tools in the United States, there are limited unbiased data quantifying the additional accuracy gained with 3D imaging. To our knowledge, our pilot trial is the first prospective study to qualitatively and quantitatively measure the additional accuracy in positioning gained with the addition of CBCT scan to robotic bronchoscopy. Several important findings from this pilot study may inform future larger prospective randomized multicenter trials powered to evaluate the diagnostic yield of ssRAB with and without CBCT, including (1) using RAB alone, the center of the target was penetrated in 26.5%, was off-center in 16.71%, was at edge in 26.5%, and was outside in 29.4% of the nodules; (2) the addition of CBCT scan to robotic bronchoscopy improved the ability to position the biopsy tool in the center of the lesion; (3) most nodules required only 1 spin to center the needle in the target; therefore, the addition of CBCT scan added minimal extra time and labor to the procedure, and the time that a ceiling-mounted CBCT spin takes is 5 to 8 seconds.

One explanation for the inaccuracy of robotic bronchoscopy alone in this study was impacted by nodule size and CT-to-body divergence, which occurred in one-half of the patients (Table 3).

**Table 3:** Procedure Characteristics (N = 98)

Characteristic	Value
Navigation time, min	4.00 (3.00-6.00)
rEBUS image	
Concentric	21 (21.4)
Eccentric	61 (62.2)
No image	16 (16.3)
Time to first needle pass, min	4.00 (2.00-5.00)
Needle size used, gauge	
21	91 (92.8)
23	3 (3.0)
19	4 (4.0)
Time to first CBCT spin, min	5.0 (4.00-7.00)
Presence of CT-to-body divergence	47 (47.9)
Presence of atelectasis after first CBCT spin	47 (47.9)
Cumulative air kerma, mGy	
Mean [SD]	91.54 [77.99]
Median (quartile 1, quartile 3)	66.1 (44.6, 113.3)
Cumulative DAP, Gy⋅cm^2^	
Mean [SD]	33.87 [25.91]
Median (quartile 1, quartile 3)	25.1 (16.9, 44.0)
Fluoroscopy time, min	
Mean [SD]	3.91 (2.16)
Median (quartile 1, quartile 3)	3.6 (2.2, 5.1)

Data are presented as No. (%), mean [SD], median (interquartile range), or as otherwise indicated. DAP = dose area product; r-EBUS = radial endobronchial ultrasound.

Preprocedural CT scans conducted during full inspiration are a requirement of robotic platforms and approximate total lung capacity. However, the procedure itself is conducted during tidal volume breathing, when lung volume approaches functional residual capacity and there is often significant atelectasis when patients breath at or below tidal volume and near the expiratory reserve volume.^8^^,^^15^ One trial demonstrated that the movement of a peripheral nodule between full inspiration and full expiration averages 17.6 mm, ranging from 6 to 30 mm in the upper lobes and 6 to 60 mm in the lower lobes.^16^ It seems that the relationship between nodules and adjacent airways may not be preserved with respiration because there is evidence that diagnostic yield of guided bronchoscopy decreases substantially in lower lobe nodules, suggesting that the relationship is not preserved with atelectasis.^18^ Although the severity of atelectasis was not an end point for this study, we recorded its occurrence and noticed that 48% of patients had evidence of atelectasis at the time of the first spin about 5 minutes into the procedure.

In addition to improving the position of the tool in relation to the center of a lesion with CBCT scan, we also recorded improvement of rEBUS images. This is likely clinically relevant because improving a view from eccentric to concentric on rEBUS has shown to increase diagnostic yield.^18^^,^^19^ However, an rEBUS view was not attainable in all nodules, likely due to targets being outside of airways in which only an eccentric or no view was possible. This was likely because the rEBUS probe tip was abutting and then deflecting off of an airway wall, which did not pass directly into the nodule (ie, a negative CT-bronchus sign^20^). Table 4 shows univariate analysis of factors that influenced center strike. rEBUS is significantly associated with center strike placement of the needle (*P* < .001). Nodules with eccentric rEBUS signal are 0.08 (95% CI, 0.02-0.23) times less likely to achieve center strike than nodules with concentric rEBUS using RAB alone. Patients with no rEBUS signal are 0.03 (95% CI, 0.001-0.16) times less likely to hit center strike than those with concentric rEBUS signal using RAB alone.

**Table 4:** Univariate Analysis of Factors Influencing Center Strike

Factors	No.	Event No.	OR	95% CI	*P* Value
Atelectasis	100	26	0.40	0.15-1.00	.051
CT-to-body divergence	95	26	0.22	0.08-0.58	.002
rEBUS view	102	28			< .001
Concentric			Reference		
Eccentric			0.08	0.02-0.23	
No Image			0.03	0.001-0.16	
Nodule size	102	28	1.15	1.05-1.28	.003
Nodule location	102	28			.21
Left lower lobe					
Left upper lobe			0.74	0.20-2.82	
Right lower lobe			0.20	0.03-0.95	
Right middle lobe			0.67	0.11-3.73	
Right upper lobe			0.36	0.09-1.47	
BMI	102	28	0.96	0.89-1.03	.24
Bronchus sign	101	28			.34
Yes			1.53	0.64-3.74	
No			Reference		
Distance from pleura axial	102	28	1.0	0.96-1.03	.74

For the univariate analysis, we categorized off-center, at edge, and outside to be 0 and center to be 1 to run the logistic model. In the presence of CT-to-body divergence, it is 0.22 (95% CI, 0.08-0.58; *P* = .002) times less likely to achieve a center strike using RAB alone. rEBUS is significantly associated with center strike placement (*P* < .001). Nodules with eccentric rEBUS signal are 0.08 (95% CI, 0.02-0.23) times less likely to achieve center strike than nodules with concentric rEBUS using RAB alone. Patients with no rEBUS signal are 0.03 (95% CI, 0.001-0.16) times less likely to hit center strike than those with concentric rEBUS signal using RAB alone. For 1-unit increase in average nodule size in diameter (mm), we expect to see about 15% (95% CI, 1.05-1.28; *P* = .003) increase in the odds of detecting center strike. Event No. = number of instances where center strike was achieved; RAB = robotic-assisted bronchoscopy; rEBUS = radial endobronchial ultrasound.

Initial biopsies were still performed without an rEBUS signal in those rare cases, even though an attempt was always made to obtain an rEBUS image. In such cases, CBCT scan showing proper bronchoscopic alignment served as an alternative confirmation when an ultrasound image could not be obtained. Often following a needle pass through an airway wall, a concentric view was achieved because the ultrasound probe could then pass through the wall previously preventing contact between the rEBUS and the nodule.

Although bronchoscopic ablation (thermal and nonthermal) of pulmonary nodules is still in the investigational stages without a clear choice as to the optimal modality, it is clear that the ability to accurately navigate to a target with a tool is at the forefront of the concerns related to these technologies.^21^^,^^22^ Despite an explosion in technology surrounding guided bronchoscopy and its ability to reach peripheral nodules, our ability to diagnose such lesions remains suboptimal.^7^ Aside from sampling tools which may need improvement,^23^^,^^24^ lower diagnostic yields published in the literature likely result from not having proper alignment of the sampling tool with the nodule as a high navigational success rate is a consistent finding throughout the literature^25^^,^^26^ (Table 5).^27^, ^28^, ^29^, ^30^, ^31^, ^32^ Therefore, before an ablative technology can be applied in clinical practice, we need to solve the dilemma of tool alignment within a target nodule to assure accurate confirmation that the ablative instrument is in position to safely and effectively treat the lesion. CBCT scan is poised to provide the solution, and this trial is a step toward validation of the accuracy of this technology in robotic bronchoscopy. Importantly, CBCT scan also provides a view of nearby anatomic structures; this could be quite useful in preventing complications, particularly when using thermal energy.^7^^,^^14^ Unlike many studies on RAB which focus on the accuracy of its diagnostic yield, we chose to define accuracy in terms of the functionality of CBCT scan when coupled with RAB. In doing so, we hoped to quantify the degree to which CBCT scan may optimize the positioning of the sampling tools, which not only may affect diagnostic yield, but is essential to understand when contemplating ablative technologies delivered using RAB. We intentionally included a qualitative and quantitative variable to determine if the tool was centered. All nodules were measured with the same approach, and because most nodules are not a true sphere but have different shapes (ie, oval, oblong, spiculated), we also assessed needle position as outside the nodule or in the center, off-center, or at the edge of the nodule. It is worth noting that CBCT scan allows for augmented fluoroscopy based on antero-posterior, sagittal, and coronal CT images. We measured distance from the center for this trial by using a single 3D view from a histogram (Fig 2). As the risk of human error was introduced, this may be viewed as a limitation of this trial. However, despite the element of manual physician measurement of distance from center and subsequent bronchoscopic adjustments, we think that incorporating qualitative measurement with quantitative measurements makes the analysis clinically meaningful in terms of diagnostic sampling and for the future, which may require the planning of ablation zones with this technique.

**Table 5:** Comparison of Study Data With Previously Published Studies Using ssRAB With CBCT Scan With Different End Points

Study	Study Design	Primary End Point	Sample Size	Target Size	Outcomes
Abdelghani et al^27^	Single center, retrospective	Compare DY of ENB and ssRAB using ceiling-mounted CBCT	ENB: 97 nodules ssRAB: 111 nodules	< 3 cm	DY with ssRAB + CBCT 89.2% vs 66% with ENB + CBCT scan
Abia-Trujillo et al^28^	Multicenter, retrospective	DY of ssRAB with and without mCBCT	192 nodules	1-2 cm	Overall DY was 85.4%, similar in both groups
Bashour et al^29^	Single center, prospective	Compare rate of TIL and DY of ssRAB alone and with mCBCT	67 nodules	1-3 cm	Improved TIL position RAB alone 34.3% vs 98.6% with CBCT scan and improved DY (29.9% vs 86.6%, respectively)
Husta et al^30^	Retrospective	Use of mobile cone beam CT with ssRAB to determine improvement with TIL relationship	102 nodules	1-3.5 cm	• In 90% of non-TIL status, conversion to TIL was possible using mCBCT • Overall DY 77%
Reisenauer et al^31^	Prospective	Improve CT-to-body divergence and DY by adding 3D imaging to ssRAB	30 nodules	1-3 cm	DY of 93% using ssRAB + mCBCT
Our study	Prospective	Place needle in center of target using ssRAB with and without ceiling-mounted CBCT	102 nodules	1-3 cm	• Improved position of biopsy tool toward the center of the lesion using ssRAB + ceiling-mounted CBCT • DY for malignant lesions 90.6%
Styrvoky et al^32^	Retrospective	Diagnostic accuracy for malignant lesions using ssRAB + CBCT scan	209 nodules	0.7-7 cm	• High diagnostic accuracy for malignant lesions (85.9%) and NPV

3D = 3-dimensional; CBCT = cone-beam computed tomography; DY = diagnostic yield; ENB = electromagnetic navigational bronchoscopy; NPV = negative predictive value; ssRAB = shape-sensing robotic-assisted bronchoscopy; TIL = tool-in-lesion.

We recognize one of the biggest limitations of this study is being single center. The ability to navigate, reach, and approach a target for biopsy may differ across different providers. It is not yet standardized how ventilation protocols are applied during peripheral bronchoscopy; however, a trend toward application of certain ventilatory parameters is being adopted. Techniques to overcome CT-to-body divergence may also vary (eg, using recruitment maneuvers when atelectasis is found, performing lateral decubitus biopsies will vary by institution and proceduralist). Our study was performed at a large academic institution by 4 different interventional pulmonologists with experience using image-guided bronchoscopy. The use of 3D imaging technology requires adequate interpreting of results followed by robotic adjustments. The ceiling-mounted CBCT with augmented fluoroscopy system is not integrated with ssRAB; therefore, our study required attention to using 2 separate systems at the time of each procedure and interpretation of results at a separate workstation. Despite this, the median time to first CBCT spin was only 5 minutes in our study. There are several factors that influence guided bronchoscopy diagnostic yield. The same factors likely apply when it comes to accuracy as defined by placing the tool in the center of the target, including nodule location, size, BMI, and presence of atelectasis. To our knowledge, there are no multicenter studies to date that have focused on our primary end point. A retrospective study by Husta et al^30^ showed the addition of 3D imaging to ssRAB improves its ability to place a biopsy tool within the lesion, but their focus was not necessarily achieving the center of the target. Another study by Bashour et al^29^ also showed that adding mobile CBCT imaging to ssRAB improved its ability to achieve tool-in-lesion and improve diagnostic yield. These studies have also been performed by experienced operators at high volume centers. Additional studies using either electromagnetic navigation bronchoscopy or ssRAB with mobile CBCT or ceiling-mounted CBCT are presented in Table 5. The table shows multiple studies with different primary end points (eg, various definitions of diagnostic yields, ability to place tool-in-lesion). Whether the results of this study can be reproducible in the community is still uncertain. Because we think being accurate is important when considering different peripheral therapeutic modalities, it is important that these interventions are initially performed at high-volume and experienced centers.

The average number of CBCT spins required for a guided bronchoscopy procedure is 2 according to published data, and the radiation exposure to the patient using CBCT scan is still within safety thresholds provided by regulatory authorities.^33^ Several studies have been published using electromagnetic navigational bronchoscopy (EMN) and robotic bronchoscopy with CBCT use. For example, Verhoeven et al^34^ reported procedural cumulative dose area product (cDAP) using EMN + ceiling-mounted CBCT ranging from 45.7 to 27.4 Gy.cm^2^ by the end of the study, which included 238 procedures. This reduction was attributed to the proceduralists becoming more experienced using CBCT scan and their ability to tailor imaging protocols with subsequent procedures.^34^ More recent studies using ssRAB + mobile CBCT scan reported a median cDAP of 17.65 Gy.cm^2^ with an average of 2 CBCT spins per case. Another study by Styrvoky et al^32^ using ssRAB with ceiling-mounted CBCT reported a mean cDAP of 22.6 Gy.cm^2^. These results are very similar to the radiation doses reported in our study using the same equipment. The radiation dose per procedure is determined by several factors including patient-related ones. A patient who is overweight (BMI 25.0-29.9 kg/m^2^) or obese (BMI > 30.0 kg/m^2^) will have a significant increase in dose area product.^35^ The mean BMI in our study cohort was 28.63 ± 6.94 kg/m^2^ with an average of 2 spins per case. Even though most of the patients fell into the overweight category, our cDAP remained similar to other published studies, as shown in Table 5, where the primary end points focused on diagnostic yields and not improving position (accuracy) within the target.

Using a standard radiation protocol, with a ceiling-mounted CBCT, we recorded a cumulative air kerma of 66.1 mGy (44.3, 113.3 mGy), a cDAP of 25.1 Gy.cm^2^ (16.9, 44.0 Gy.cm^2^), and a mean fluoroscopy time of 3.6 minutes (2.2, 5.1 min), similar to other published data.^19^^,^^27^^,^^33^^,^^36^ It is important to note that our primary end point did not translate into having an increased cDAP. The CBCT equipment was fully operated by the bronchoscopist during each procedure, and there were no complications related to the use of CBCT scan.

## Interpretation

In this pilot study, the addition of CBCT scan to RAB improved the position of a biopsy tool in relation to the center of a pulmonary nodule with little extra time, and with acceptable radiation exposure. The accuracy, stability, and ability to negotiate different positions within a target provided by shape-sensing RAB is complemented by CBCT adjustments when necessary. To our knowledge, this study is the first to quantify the accuracy achieved using both technologies, which may translate into reliability to perform diagnostic and potentially future therapeutic interventions in guided bronchoscopy.

## Funding/Support

This research study was partially funded by Philips.

## Financial/Nonfinancial Disclosures

A. E. R. and C. G. reports consulting relationship with Intuitive Surgical Inc; J. C. H. reports consulting relationship with Boehringer Ingelheim; N. P. reports consulting relationship with Olympus America Inc. None declared: J. P., M. W., J. P. and J. M.

## Supplementary Data

e-Online Data
