# Supplementary material for: The Additional Accuracy Gained by Cone Beam CT in Shape-Sensing Robotic Bronchoscopy
Source: CHEST Pulm. 2025 Aug 7;3(4):100203. doi: 10.1016/j.chpulm.2025.100203 (PMC13418395; doi:10.1016/j.chpulm.2025.100203)
Supplement: e-Online Data [file mmc1.docx]

**SUPPLEMENTAL MATERIAL**


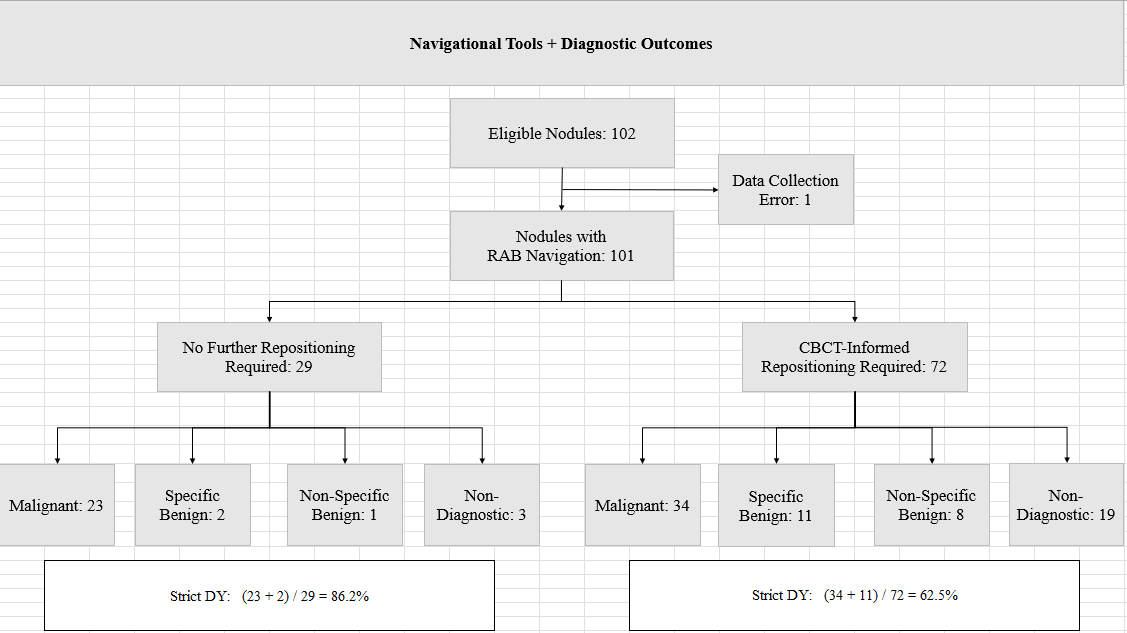


Comparison of diagnostic yields RAB alone vs RAB + CBCT, however the sample size for RAB alone is very small. Nodules that did not require further repositioning had a higher DY of 86.2%, compared to 62.5% for those nodules that did require CBCT-informed repositioning. Note that this higher DY could just indicate that these 29 nodules were in easier locations to biopsy. This result cannot be used to conclude that biopsy using RAB alone is necessarily superior to RAB + CBCT. Larger samples are needed in both groups.
